# (*Z*)-1-(2-Hy­droxy­eth­yl)-4-(2-meth­oxy­benzyl­idene)-2-methyl-1*H*-imidazol-5(4*H*)-one

**DOI:** 10.1107/S1600536813007770

**Published:** 2013-03-28

**Authors:** Hongyi Wu, Weihua Wang, Edwin H. Walker, Frank R. Fronczek

**Affiliations:** a Department of Chemistry, Southern University, Baton Rouge, LA 70813, USA; b Department of Chemistry, Louisiana State University, Baton Rouge, LA 70803-1804, USA

## Abstract

In the title compound, C_14_H_16_N_2_O_3_, an analog of the chromophore in green fluorescent protein, the meth­oxy­phenyl substituent and the imidazole N adopt a *Z* conformation with respect to the C=C bond. Aside from the hy­droxy­ethyl group, the mol­ecule is approximately planar, with the five- and six-membered ring planes forming a dihedral angle of 9.3 (1)°. An intra­molecular C—H⋯N contact occurs. In the crystal, O—H⋯N hydrogen bonds link the mol­ecules, forming chains along the *b*-axis direction. C—H⋯O hydrogen bonds are also observed.

## Related literature

For background to green fluorescent protein, see: Shimomura *et al.* (1962 ▶); Shimomura (2009 ▶); Remington (2006 ▶); Tsien (1998 ▶); Chalfie *et al.* (1994 ▶); Prasher *et al.* (1992 ▶). For the synthesis, see: Yampolsky *et al.* (2005 ▶); Bailly *et al.*(2004 ▶); Wenge & Wagenknecht (2011 ▶). For related structures, see: Naumov *et al.* (2010 ▶); Bhattacharjya *et al.* (2005 ▶); Oshimi *et al.* (2002 ▶); Dong *et al.* (2009 ▶). For Bijvoet pair analysis, see: Hooft *et al.* (2008 ▶).
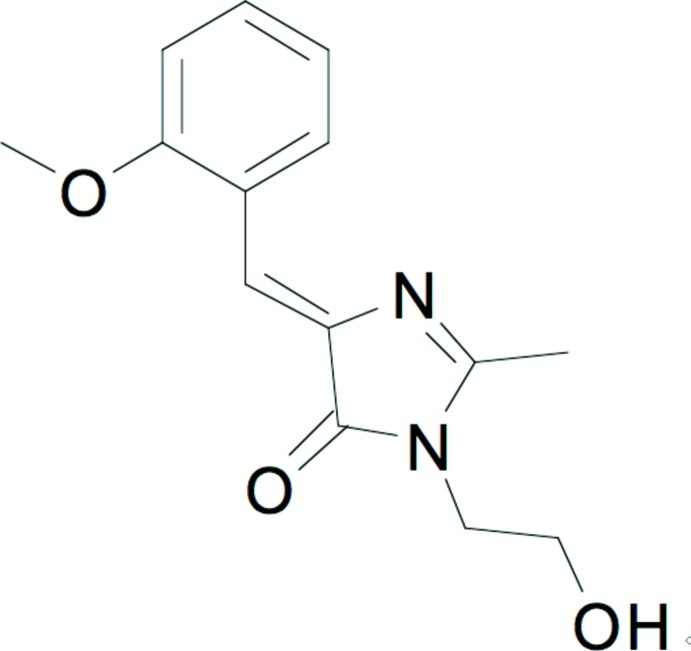


## Experimental

### 

#### Crystal data

C_14_H_16_N_2_O_3_
*M* *_r_* = 260.29
Monoclinic, 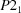

*a* = 9.2188 (5) Å
*b* = 7.2767 (4) Å
*c* = 9.5620 (5) Å
β = 93.625 (6)°
*V* = 640.16 (6) Å^3^
*Z* = 2
Mo *K*α radiation
μ = 0.10 mm^−1^
*T* = 90 K
0.35 × 0.25 × 0.17 mm

#### Data collection

Bruker Kappa APEXII DUO CCD diffractometer
Absorption correction: multi-scan (*SADABS*; Sheldrick, 2004 ▶) *T*_min_ = 0.967, *T*_max_ = 0.984
9385 measured reflections
4943 independent reflections
4720 reflections with *I* > 2σ(*I*)
*R*_int_ = 0.017

#### Refinement

*R*[*F* ^2^ > 2σ(*F* ^2^)] = 0.032
*wR*(*F* ^2^) = 0.087
*S* = 1.06
4943 reflections
177 parameters
1 restraint
H atoms treated by a mixture of independent and constrained refinement
Δρ_max_ = 0.42 e Å^−3^
Δρ_min_ = −0.24 e Å^−3^
Absolute structure: Flack (1983 ▶), 1605 Friedel pairs
Flack parameter: −0.9 (5)

### 

Data collection: *APEX2* (Bruker, 2006 ▶); cell refinement: *SAINT* (Bruker, 2006 ▶); data reduction: *SAINT*; program(s) used to solve structure: *SHELXS97* (Sheldrick, 2008 ▶); program(s) used to refine structure: *SHELXL97* (Sheldrick, 2008 ▶); molecular graphics: *ORTEP-3 for Windows* (Farrugia, 2012 ▶); software used to prepare material for publication: *SHELXL97* (Sheldrick, 2008 ▶).

## Supplementary Material

Crystal structure: contains datablock(s) global, I. DOI: 10.1107/S1600536813007770/sj5304sup1.cif

Structure factors: contains datablock(s) I. DOI: 10.1107/S1600536813007770/sj5304Isup2.hkl

Supplementary material file. DOI: 10.1107/S1600536813007770/sj5304Isup3.cml

Additional supplementary materials:  crystallographic information; 3D view; checkCIF report

## Figures and Tables

**Table 1:** Hydrogen-bond geometry (Å, °)

*D*—H⋯*A*	*D*—H	H⋯*A*	*D*⋯*A*	*D*—H⋯*A*
O3—H3*O*⋯N2^i^	0.879 (16)	2.001 (16)	2.8771 (9)	174.2 (15)
C4—H4*B*⋯O1^ii^	0.99	2.54	3.2993 (10)	133
C9—H9⋯N2	0.95	2.52	3.1729 (10)	126
C14—H14*A*⋯O1^iii^	0.98	2.52	3.3475 (12)	141

Symmetry codes: (i) 
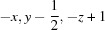
; (ii) 
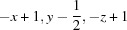
; (iii) 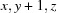.

**Fig. 1.**
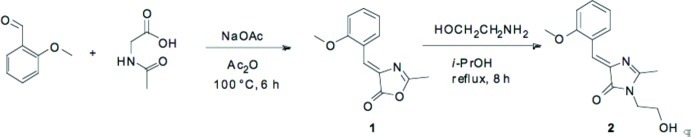
Scheme showing the synthesis.

**Fig. 2.**
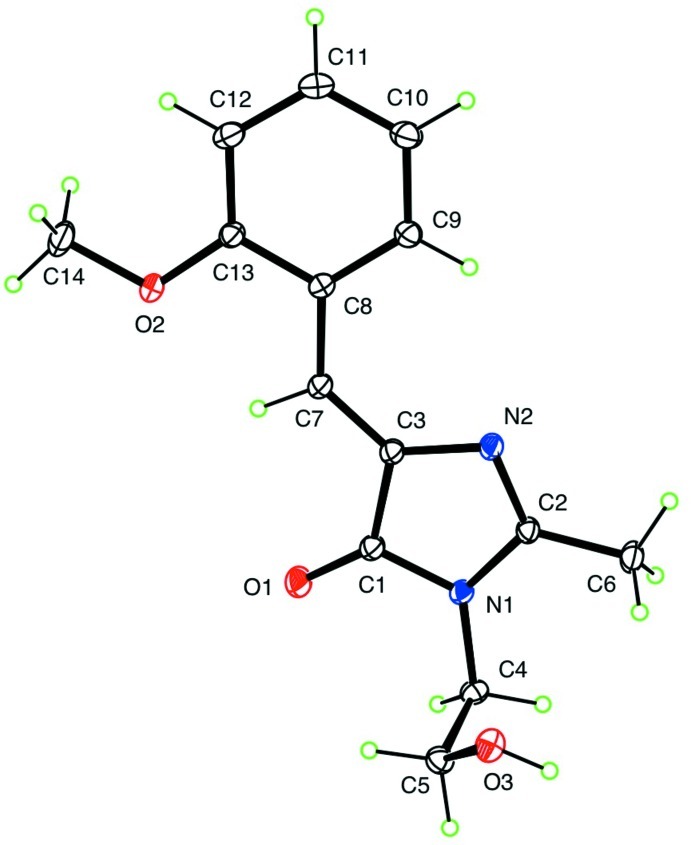
Ellipsoids at the 50% level, with H atoms having arbitrary radius.

## supplementary crystallographic information

### Comment

The title compound is an analog of the chromophore in green fluorescent protein (GFP). GFP was first identified and separated from the jellyfish *Aequorea victoria* in the 1960s. Since then, GFP has found broad use in many areas of science and medicine, especially as fluorescent labels for cell biology and biotechnology. (Shimomura *et al.*, 1962; Shimomura, 2009; Remington, 2006; Tsien, 1998; Chalfie *et al.*, 1994) Though the GFP is a protein composed of more than two hundred amino acid residues, its chromophore (*p*-hydroxybenzylidene-imidazol-5-one) is relatively small. In nature, the GFP chromophore is formed *via* the sequential cyclization-oxidation-dehydration of the Ser^65^—Tyr^66^—Gly^67^ tripeptide motif. (Prasher *et al.*, 1992)

Preparation of the title compound starts with the Erlenmeyer azlactone synthesis, which involves the condensation of hippuric acid derivatives with aromatic aldehydes (Yampolsky *et al.*, 2005; Bailly *et al.*, 2004), Fig. 1. Further reaction of the resulting azlactone with ethanolamine leads to the formation of the title compound. We report the crystal structure of the compound here, which shows the compound has a *Z*-configuration. The compound is used as a model compound in our study of E, *Z*-isomerization of chromophores in fluorescent proteins.

The structure of the molecule is shown in Fig. 2. The *Z* configuration is evidenced by the torsion angle N2–C3–C7–C8 3.33 (14)° about the central double bond. The hydroxyethyl group is twisted away from coplanarity with the rest of the molecule (C1–N1–C4–C5 torsion angle -74.77 (9)°), but otherwise, the molecule is relatively planar, with the phenyl and imidazole rings forming a dihedral angle of 9.3 (1)°.

The OH group O3 forms a near-linear intermolecular hydrogen bond to the imidazole nitrogen atom N2 (at -x, y-1/2, 1-z), forming chains in the *b* direction, propagated by the screw axis. Several intermolecular C–H···O hydrogen bonds and an intramolecular C–H···N contact also exist, as given in Table 1.

### Experimental

A mixture of *o*-anisaldehyde (6.95 g, 50.0 mmol), *N*-acetylglycine (5.97 g, 50.5 mmol) and anhydrous sodium acetate (4.35 g, 52.5 mmol) were dissolved in 20 ml acetic anhydride. The mixture was stirred at 100° C for 6 h. Upon completion, the reaction mixture was cooled to room temperature. After the addition of 10 ml ice-cold water, the resulting precipitate was collected by gravity filtration. The filtrand was then washed three times with ice-cold water and dried in vacuum, yielding (1) as a yellow powder (8.41 g, 64.7%). Title compound (2) was synthesized by reacting (1) with ethanolamine in 2-propanol. To a suspension of compound (1) (3.30 g, 15.2 mmol) in dried 2-propanol (30 ml), ethanolamine (1.14 ml, 18.8 mmol) was added gradually. The reaction mixture was refluxed for 8 h. The solvent was then removed under vacuo. The crude product was recrystallized from a n-butanol/diethyl ether (1/1, *v*/*v*) mixture. Yellow crystals of the title compound (2) were obtained in a yield of 2.77 g (58%). The sample crystal was grown by evaporation from methanol.

FT—IR Characterization (cm^-1^): 3237, 2944, 1711, 1635, 1423, 1256

NMR Characterization: ^1^H NMR (400 MHz, CD_3_Cl): δ 2.39 (s, 3 H, CH_3_C), 3.74 (t, J = 5.9 Hz, 2H, CH_2_), 3.81 (t, J=5.9 Hz, 2H, CH_2_),3.89 (s, 3 H, CH_3_O), 6.89 (m, 1 H, ArH), 7.02(m, 1 H, ArH), 7.35 (m, 1H, ArH), 7.67 (s, 1 H, HC═C), 8.68 (m, 1 H, ArH); ^13^C NMR (100 MHz, CD_3_Cl): δ = 15.9(CH_3_C), 43.7 (CH_2_), 55.6(CH_3_O), 62.0 (CH_2_), 110.7 (ArCH), 120.9 (ArCH), 122.0 (HC═C),123.1 (ArCC), 131.8 (ArCH), 132.9 (ArCH), 137.7 (HC═C), 159.2(C—O), 162.3 (C═N), 171.3 (C═O).

### Refinement

H atoms on C were located from difference maps, but were placed in idealized positions with C—H distance 0.95 - 0.99 Å, depending on atom type. A torsional parameter was refined for each methyl group. Coordinates for the hydroxy H atom were refined. *U*_iso_ for H were assigned as 1.2 times *U*_eq_ of the attached atoms (1.5 for methyl and OH). Refinement of the Flack (1983) parameter was inconclusive; however, analysis of the Bijvoet pairs by the Hooft *et al.* (2008) method yielded a P2(true) value of 1.000. Although the molecule is not inherently chiral, we consider the reported coordinates to likely represent the correct absolute structure of the crystal studied, and the pairs were kept separate in the refinement.

### Crystal data

**Table:**

C_14_H_16_N_2_O_3_	*F*(000) = 276
*M**_r_* = 260.29	*D*_x_ = 1.350 Mg m^−^^3^
Monoclinic, *P*2_1_	Mo *K*α radiation, λ = 0.71073 Å
Hall symbol: P 2yb	Cell parameters from 6251 reflections
*a* = 9.2188 (5) Å	θ = 3.2–37.7°
*b* = 7.2767 (4) Å	µ = 0.10 mm^−^^1^
*c* = 9.5620 (5) Å	*T* = 90 K
β = 93.625 (6)°	Needle, yellow
*V* = 640.16 (6) Å^3^	0.35 × 0.25 × 0.17 mm
*Z* = 2	

### Data collection

**Table:**

Bruker Kappa APEXII DUO CCD diffractometer	4943 independent reflections
Radiation source: fine-focus sealed tube	4720 reflections with *I* > 2σ(*I*)
TRIUMPH curved graphite monochromator	*R*_int_ = 0.017
φ and ω scans	θ_max_ = 37.8°, θ_min_ = 3.2°
Absorption correction: multi-scan (*SADABS*; Sheldrick, 2004)	*h* = −15→13
*T*_min_ = 0.967, *T*_max_ = 0.984	*k* = −8→12
9385 measured reflections	*l* = −15→15

### Refinement

**Table:**

Refinement on *F*^2^	Secondary atom site location: difference Fourier map
Least-squares matrix: full	Hydrogen site location: inferred from neighbouring sites
*R*[*F*^2^ > 2σ(*F*^2^)] = 0.032	H atoms treated by a mixture of independent and constrained refinement
*wR*(*F*^2^) = 0.087	*w* = 1/[σ^2^(*F*_o_^2^) + (0.0603*P*)^2^ + 0.0209*P*] where *P* = (*F*_o_^2^ + 2*F*_c_^2^)/3
*S* = 1.06	(Δ/σ)_max_ < 0.001
4943 reflections	Δρ_max_ = 0.42 e Å^−^^3^
177 parameters	Δρ_min_ = −0.24 e Å^−^^3^
1 restraint	Absolute structure: Flack (1983), 1605 Friedel pairs
Primary atom site location: structure-invariant direct methods	Flack parameter: −0.9 (5)

### Special details

**Table:**

Geometry. All e.s.d.'s (except the e.s.d. in the dihedral angle between two l.s. planes) are estimated using the full covariance matrix. The cell e.s.d.'s are taken into account individually in the estimation of e.s.d.'s in distances, angles and torsion angles; correlations between e.s.d.'s in cell parameters are only used when they are defined by crystal symmetry. An approximate (isotropic) treatment of cell e.s.d.'s is used for estimating e.s.d.'s involving l.s. planes.
Refinement. Refinement of *F*^2^ against ALL reflections. The weighted *R*-factor *wR* and goodness of fit *S* are based on *F*^2^, conventional *R*-factors *R* are based on *F*, with *F* set to zero for negative *F*^2^. The threshold expression of *F*^2^ > σ(*F*^2^) is used only for calculating *R*-factors(gt) *etc*. and is not relevant to the choice of reflections for refinement. *R*-factors based on *F*^2^ are statistically about twice as large as those based on *F*, and *R*- factors based on ALL data will be even larger.

### Fractional atomic coordinates and isotropic or equivalent isotropic displacement parameters (Å^2^)

**Table:**

	*x*	*y*	*z*	*U*_iso_*/*U*_eq_	
O1	0.46857 (7)	0.27870 (9)	0.59877 (7)	0.01987 (12)	
O2	0.52843 (7)	0.87448 (9)	0.79824 (7)	0.01835 (11)	
O3	0.11188 (6)	0.14749 (9)	0.32174 (6)	0.01679 (11)	
H3O	0.0433 (16)	0.064 (2)	0.3128 (15)	0.025*	
N1	0.23432 (7)	0.16329 (9)	0.60614 (7)	0.01255 (10)	
N2	0.12395 (7)	0.39039 (9)	0.72030 (7)	0.01344 (11)	
C1	0.34363 (8)	0.29285 (10)	0.63215 (8)	0.01374 (12)	
C2	0.10888 (8)	0.22969 (11)	0.65921 (7)	0.01237 (11)	
C3	0.26991 (8)	0.44057 (10)	0.70824 (8)	0.01307 (12)	
C4	0.25234 (8)	0.00285 (10)	0.51780 (8)	0.01355 (12)	
H4A	0.1748	−0.0877	0.5335	0.016*	
H4B	0.3473	−0.0558	0.5431	0.016*	
C5	0.24500 (8)	0.05885 (12)	0.36415 (8)	0.01478 (12)	
H5A	0.3268	0.1428	0.3481	0.018*	
H5B	0.2563	−0.0519	0.3057	0.018*	
C6	−0.02821 (9)	0.12268 (11)	0.64852 (9)	0.01714 (13)	
H6A	−0.1019	0.1860	0.7002	0.026*	
H6B	−0.0108	0.0001	0.6885	0.026*	
H6C	−0.0626	0.1112	0.5498	0.026*	
C7	0.34421 (8)	0.59176 (10)	0.75311 (8)	0.01367 (12)	
H7	0.4419	0.5965	0.7272	0.016*	
C8	0.29964 (8)	0.74799 (10)	0.83426 (7)	0.01242 (11)	
C9	0.16563 (8)	0.75856 (11)	0.89605 (8)	0.01475 (12)	
H9	0.0969	0.6622	0.8807	0.018*	
C10	0.13176 (9)	0.90681 (13)	0.97897 (8)	0.01816 (14)	
H10	0.0408	0.9115	1.0203	0.022*	
C11	0.23173 (9)	1.04896 (12)	1.00136 (8)	0.01849 (14)	
H11	0.2081	1.1511	1.0575	0.022*	
C12	0.36595 (9)	1.04289 (11)	0.94234 (8)	0.01678 (13)	
H12	0.4338	1.1400	0.9584	0.020*	
C13	0.39992 (8)	0.89311 (10)	0.85941 (7)	0.01325 (11)	
C14	0.64199 (10)	1.00233 (13)	0.83403 (11)	0.02252 (16)	
H14A	0.6138	1.1243	0.7984	0.034*	
H14B	0.7312	0.9629	0.7921	0.034*	
H14C	0.6590	1.0076	0.9362	0.034*	

### Atomic displacement parameters (Å^2^)

**Table:**

	*U*^11^	*U*^22^	*U*^33^	*U*^12^	*U*^13^	*U*^23^
O1	0.0123 (2)	0.0188 (3)	0.0289 (3)	−0.0010 (2)	0.0046 (2)	−0.0056 (2)
O2	0.0164 (2)	0.0172 (3)	0.0220 (2)	−0.0070 (2)	0.00527 (19)	−0.0056 (2)
O3	0.0147 (2)	0.0152 (2)	0.0201 (2)	0.00069 (19)	−0.00168 (18)	0.0018 (2)
N1	0.0115 (2)	0.0103 (2)	0.0159 (2)	−0.00049 (19)	0.00164 (18)	−0.0022 (2)
N2	0.0120 (2)	0.0111 (2)	0.0174 (2)	−0.00118 (19)	0.00251 (19)	−0.0013 (2)
C1	0.0126 (3)	0.0119 (3)	0.0168 (3)	−0.0009 (2)	0.0014 (2)	−0.0022 (2)
C2	0.0119 (3)	0.0103 (2)	0.0150 (3)	−0.0010 (2)	0.0021 (2)	0.0005 (2)
C3	0.0120 (3)	0.0109 (3)	0.0164 (3)	−0.0007 (2)	0.0021 (2)	−0.0018 (2)
C4	0.0156 (3)	0.0096 (3)	0.0155 (3)	0.0011 (2)	0.0008 (2)	−0.0015 (2)
C5	0.0141 (3)	0.0154 (3)	0.0149 (3)	0.0009 (2)	0.0013 (2)	−0.0004 (2)
C6	0.0132 (3)	0.0135 (3)	0.0250 (3)	−0.0035 (2)	0.0034 (2)	−0.0009 (3)
C7	0.0129 (3)	0.0116 (3)	0.0167 (3)	−0.0018 (2)	0.0020 (2)	−0.0026 (2)
C8	0.0134 (3)	0.0109 (3)	0.0129 (2)	−0.0003 (2)	0.0002 (2)	−0.0008 (2)
C9	0.0132 (3)	0.0157 (3)	0.0154 (3)	0.0001 (2)	0.0012 (2)	−0.0022 (2)
C10	0.0176 (3)	0.0191 (3)	0.0179 (3)	0.0020 (3)	0.0029 (2)	−0.0041 (3)
C11	0.0224 (3)	0.0162 (3)	0.0169 (3)	0.0018 (3)	0.0012 (2)	−0.0048 (3)
C12	0.0207 (3)	0.0132 (3)	0.0162 (3)	−0.0015 (3)	0.0000 (2)	−0.0028 (2)
C13	0.0149 (3)	0.0117 (3)	0.0132 (2)	−0.0019 (2)	0.0009 (2)	−0.0006 (2)
C14	0.0187 (3)	0.0176 (3)	0.0313 (4)	−0.0080 (3)	0.0022 (3)	−0.0031 (3)

### Geometric parameters (Å, º)

**Table:**

O1—C1	1.2188 (9)	C6—H6A	0.9800
O2—C13	1.3608 (9)	C6—H6B	0.9800
O2—C14	1.4259 (10)	C6—H6C	0.9800
O3—C5	1.4225 (10)	C7—C8	1.4504 (10)
O3—H3O	0.879 (16)	C7—H7	0.9500
N1—C2	1.3794 (9)	C8—C9	1.4048 (10)
N1—C1	1.3908 (10)	C8—C13	1.4141 (10)
N1—C4	1.4566 (10)	C9—C10	1.3861 (11)
N2—C2	1.3105 (10)	C9—H9	0.9500
N2—C3	1.4061 (10)	C10—C11	1.3929 (12)
C1—C3	1.4863 (10)	C10—H10	0.9500
C2—C6	1.4825 (11)	C11—C12	1.3928 (12)
C3—C7	1.3515 (11)	C11—H11	0.9500
C4—C5	1.5221 (11)	C12—C13	1.3950 (11)
C4—H4A	0.9900	C12—H12	0.9500
C4—H4B	0.9900	C14—H14A	0.9800
C5—H5A	0.9900	C14—H14B	0.9800
C5—H5B	0.9900	C14—H14C	0.9800
			
C13—O2—C14	118.54 (7)	C2—C6—H6C	109.5
C5—O3—H3O	108.3 (10)	H6A—C6—H6C	109.5
C2—N1—C1	108.16 (6)	H6B—C6—H6C	109.5
C2—N1—C4	128.54 (6)	C3—C7—C8	130.76 (7)
C1—N1—C4	122.62 (6)	C3—C7—H7	114.6
C2—N2—C3	105.67 (6)	C8—C7—H7	114.6
O1—C1—N1	125.59 (7)	C9—C8—C13	118.06 (7)
O1—C1—C3	131.16 (7)	C9—C8—C7	123.67 (7)
N1—C1—C3	103.25 (6)	C13—C8—C7	118.17 (6)
N2—C2—N1	114.13 (6)	C10—C9—C8	121.27 (7)
N2—C2—C6	124.42 (7)	C10—C9—H9	119.4
N1—C2—C6	121.44 (7)	C8—C9—H9	119.4
C7—C3—N2	130.83 (7)	C9—C10—C11	119.67 (7)
C7—C3—C1	120.39 (7)	C9—C10—H10	120.2
N2—C3—C1	108.78 (6)	C11—C10—H10	120.2
N1—C4—C5	110.21 (6)	C12—C11—C10	120.68 (7)
N1—C4—H4A	109.6	C12—C11—H11	119.7
C5—C4—H4A	109.6	C10—C11—H11	119.7
N1—C4—H4B	109.6	C11—C12—C13	119.49 (7)
C5—C4—H4B	109.6	C11—C12—H12	120.3
H4A—C4—H4B	108.1	C13—C12—H12	120.3
O3—C5—C4	112.42 (6)	O2—C13—C12	123.71 (7)
O3—C5—H5A	109.1	O2—C13—C8	115.47 (6)
C4—C5—H5A	109.1	C12—C13—C8	120.81 (7)
O3—C5—H5B	109.1	O2—C14—H14A	109.5
C4—C5—H5B	109.1	O2—C14—H14B	109.5
H5A—C5—H5B	107.9	H14A—C14—H14B	109.5
C2—C6—H6A	109.5	O2—C14—H14C	109.5
C2—C6—H6B	109.5	H14A—C14—H14C	109.5
H6A—C6—H6B	109.5	H14B—C14—H14C	109.5
			
C2—N1—C1—O1	−179.73 (8)	N1—C4—C5—O3	−58.15 (8)
C4—N1—C1—O1	−8.46 (12)	N2—C3—C7—C8	3.33 (14)
C2—N1—C1—C3	0.98 (8)	C1—C3—C7—C8	−176.57 (7)
C4—N1—C1—C3	172.26 (6)	C3—C7—C8—C9	7.28 (13)
C3—N2—C2—N1	0.45 (8)	C3—C7—C8—C13	−176.50 (8)
C3—N2—C2—C6	179.24 (7)	C13—C8—C9—C10	0.22 (11)
C1—N1—C2—N2	−0.96 (9)	C7—C8—C9—C10	176.44 (7)
C4—N1—C2—N2	−171.57 (7)	C8—C9—C10—C11	0.31 (12)
C1—N1—C2—C6	−179.80 (7)	C9—C10—C11—C12	−0.59 (13)
C4—N1—C2—C6	9.60 (11)	C10—C11—C12—C13	0.33 (12)
C2—N2—C3—C7	−179.70 (8)	C14—O2—C13—C12	8.09 (11)
C2—N2—C3—C1	0.21 (8)	C14—O2—C13—C8	−171.68 (7)
O1—C1—C3—C7	−0.05 (13)	C11—C12—C13—O2	−179.54 (7)
N1—C1—C3—C7	179.18 (7)	C11—C12—C13—C8	0.22 (11)
O1—C1—C3—N2	−179.97 (9)	C9—C8—C13—O2	179.29 (7)
N1—C1—C3—N2	−0.74 (8)	C7—C8—C13—O2	2.86 (10)
C2—N1—C4—C5	94.61 (9)	C9—C8—C13—C12	−0.49 (11)
C1—N1—C4—C5	−74.77 (9)	C7—C8—C13—C12	−176.92 (7)

### Hydrogen-bond geometry (Å, º)

**Table:**

*D*—H···*A*	*D*—H	H···*A*	*D*···*A*	*D*—H···*A*
O3—H3*O*···N2^i^	0.879 (16)	2.001 (16)	2.8771 (9)	174.2 (15)
C4—H4*B*···O1^ii^	0.99	2.54	3.2993 (10)	133
C9—H9···N2	0.95	2.52	3.1729 (10)	126
C14—H14*A*···O1^iii^	0.98	2.52	3.3475 (12)	141

Symmetry codes: (i) −*x*, *y*−1/2, −*z*+1; (ii) −*x*+1, *y*−1/2, −*z*+1; (iii) *x*, *y*+1, *z*.
